# Occupational Exposure to Ultrafine Particles among Airport Employees - Combining Personal Monitoring and Global Positioning System

**DOI:** 10.1371/journal.pone.0106671

**Published:** 2014-09-09

**Authors:** Karina Lauenborg Møller, Lau Caspar Thygesen, Jasper Schipperijn, Steffen Loft, Jens Peter Bonde, Sigurd Mikkelsen, Charlotte Brauer

**Affiliations:** 1 National Institute of Public Health, University of Southern Denmark, Copenhagen, Denmark; 2 Institute of Sports Science and Clinical Biomechanics, University of Southern Denmark, Odense, Denmark; 3 Section of Environmental Health, Department of Public Health, University of Copenhagen, Copenhagen, Denmark; 4 Department of Occupational and Environmental Medicine, Copenhagen University Hospital Bispebjerg, Copenhagen, Denmark; Indian Institute of Toxicology Research, India

## Abstract

**Background:**

Exposure to ultrafine particles (UFP) has been linked to cardiovascular and lung diseases. Combustion of jet fuel and diesel powered handling equipment emit UFP resulting in potentially high exposure levels among employees working at airports. High levels of UFP have been reported at several airports, especially on the apron, but knowledge on individual exposure profiles among different occupational groups working at an airport is lacking.

**Purpose:**

The aim of this study was to compare personal exposure to UFP among five different occupational groups working at Copenhagen Airport (CPH).

**Method:**

30 employees from five different occupational groups (baggage handlers, catering drivers, cleaning staff and airside and landside security) at CPH were instructed to wear a personal monitor of particle number concentration in real time and a GPS device. The measurements were carried out on 8 days distributed over two weeks in October 2012. The overall differences between the groups were assessed using linear mixed model.

**Results:**

Data showed significant differences in exposure levels among the groups when adjusted for variation within individuals and for effect of time and date (p<0.01). Baggage handlers were exposed to 7 times higher average concentrations (geometric mean, GM: 37×10^3^ UFP/cm^3^, 95% CI: 25–55×10^3^ UFP/cm^3^) than employees mainly working indoors (GM: 5×10^3^ UFP/cm^3^, 95% CI: 2–11×10^3^ UFP/cm^3^). Furthermore, catering drivers, cleaning staff and airside security were exposed to intermediate concentrations (GM: 12 to 20×10^3^ UFP/cm^3^).

**Conclusion:**

The study demonstrates a strong gradient of exposure to UFP in ambient air across occupational groups of airport employees.

## Background

There is a growing scientific consensus that particulate air pollution in urban environments may increase the risk of ischemic heart disease, chronic respiratory disease and possibly several types of cancer [1]–[5]. However, there is still uncertainty about the significance of an association between occupational exposure to particulate air pollution and related health effects [6]. In recent years the scientific interest has moved from mass concentration to the number of ultrafine particles (UFP; diameter ≤100 nm) [7], because of their high alveolar deposition fraction, insolubility, large surface area, toxic constituents and potential ability to penetrate into the blood vessels [1], [8], [9].

At airports combustion of jet fuel and diesel from aircraft and handling equipment emits large numbers of UFP and employees working at an airport may be exposed to high levels, especially ground personnel working on the apron near the aircraft [10]. Stationary measurements carried out by the Danish Centre for Environment and Energy in 2010 showed high concentrations of UFP at Copenhagen Airport (CPH) with an average concentration from August to October 2010 of 43×10^3^ particles/cm^3^ in the size 6–700 nm. In comparison they found an average concentration about 10×10^3^ particles/cm^3^ (6–700 nm) at the most polluted area in Copenhagen city centre (H.C. Andersens Boulevard) in the same period [10]. About 90% of the measured particles (6–700 nm) were UFP in the size-fraction from 6–40 nm [10].

Only two studies have investigated adverse health effects related to occupational exposure to UFP at civil airports [11], [12]. Both studies supported an association between occupational exposure to UFP at an airport and adverse respiratory health effects. Because high concentrations of UFP are correlated to the apron, the exposure to UFP is thought to vary considerably for different groups of employees [10]. However, to date the exposure classification is based upon job title classifications and knowledge about high levels of air pollution at the airport or around the airplane, but the relationship between individual exposure to air pollution and job title is not known [11], [12]. Without quantitative data on occupational exposure to UFP compared across job functions the identification of health risks for different occupational groups at the airport could be inaccurate [13]. Further, the exposure profile at an airport is rather complex with a mixture of particles and gases from jet engines and diesel-powered vehicles. An essential component in understanding the exposure profile is therefore knowledge about where employees spend their working time in combination with UFP concentrations at these locations, as the particle concentration varies from place to place [14]. It is therefore questionable whether exposure estimation through knowledge from stationary measurements and job titles describe the real differences between exposure of different groups of employees working at airports [14].

The use of Global Positioning Systems (GPS) in health research has allowed researchers to accurately track the location of participants [15]. The majority of health literature including GPS data focuses on physical activity, but also travel routes are investigated since information on location can be linked to corresponding data on air pollution allowing for identification of low and high air pollution exposure routes [16]–[18]. Studies have shown that GPS measurements give more reliable results compared to activity diaries [15]. However, no previous study has investigated the exposure profile among different occupational groups at an airport based on the combined assessment of location of the employees at work and personal monitoring of UFP concentrations.

The aim of this study was to compare the personal exposure of UFP among five different occupational groups working at CPH.

## Method

### Ethics Statement

This study is a part of a project addressing occupational exposure to particulate matter and related health effects in Copenhagen Airport, Kastrup. The Danish Data Protection Agency has notified this project (Journal no: 2012-41-0199). Further the Danish National Committee on Health Research Ethics was contacted (Journal no: H-3-2012-027), but according to Danish Law this project did not require approval by an ethical committee, since this is only mandatory for projects using biological material. All participants included in the study gave written informed consent.

### Location

Data used in this paper were gathered at CPH. CPH is located 8 km from the city center of Copenhagen and is the largest airport in Denmark, with an area of 12.4 km^2^ and is the daily workplace for approximately 22 000 employees. In 2012, the total number of international and domestic flights was close to 250 000. The apron is the area at the airport where aircrafts are parked, unloaded and loaded, refueled or boarded. At CPH most of the apron is facing south-west, and with a typical wind direction from the south-west in Denmark, wind speed and direction can have a significant impact on the number concentration of UFP.

### Participants

Personal exposure measurements were carried out for employees from five different occupational groups:

1. Baggage handlers: Assigned to aircraft procedures on the apron such as luggage loading and unloading, both inside and outside the baggage compartment. Further this group is assigned to push the aircraft to the taxi way using a push back tractor.
2. Catering drivers: Assigned to load and unload food and drinks to and from the aircraft. This group went into the aircraft from the apron with a diesel powered high loader.
3. Cleaning staff: Assigned for aircraft cabin cleaning. This group went into the aircraft from the apron with a diesel powered high loader or lorry. The front and rear doors in the aircraft were open during servicing.
4. Airside security: Assigned to security service at the security restricted area and to patrol by vehicle on the apron, gates and along fence lines and buildings.
5. Landside security: Assigned to security service inside the terminal building.

Inclusion of these five occupational groups enabled comparison between occupational groups working at CPH with long-term and intermittent stay on the apron. 40 voluntary employees agreed to participate in the study, 8 from each occupational group. As a result of logistic and instrumental problems measurements could only be carried out for 30 employees. We prioritized measurements in occupational groups with outdoor work, and reduced the number of measurements for security landside (working indoors) to two measurements.

### Instruments and measurements

The personal UFP exposure measurements were collected by mobile UFP monitors (NanoTracer, Phillips). The NanoTracer is a portable electronic UFP monitoring device that enables measurements of airborne particles between 10 and 300 nm and is equipped with an internal rechargeable battery that lasts seven hours on a single charge [19]. The measurements were carried out using the NanoTracers advanced mode which allows recording both particle size and concentration of UFP/cm^3^ with a sampling interval of 16 seconds. In the device manual the accuracy is specified to be ±1 500 UFP/cm^3^ for particle concentration and ±10 nm for particle diameter [19]. The performances of three of the NanoTracer instruments used for the present study were recently compared with parallel measurements by a Scanning Mobility Particle Sizer [20]. Except for particles with diameters exceeding 125 nm, which appear to be overestimated by the NanoTracers there was good agreement between the instruments [20].

Location information was recorded using the QStarz BT-Q1000XT GPS tracking unit. The Qstarz GPS unit has a relatively high location accuracy with 50% of the measurements located inside a radius of 3 meters, and good inter-unit reliability compared to other units [21]. The GPS units were set-up using the open source BT747 software (www.bt747.org). Units were configured to log data every second, but an unknown software error resulted in a few random GPS units logging at 5 or 15 seconds instead of 1. This error was corrected when merging the data from the two instruments. The GPS units were set to record latitude and longitude (i.e. position), altitude, speed, Positional Dilution of Precision (PDOP), and the number of satellites used and in view (used to calculate satellite to noise ratio). PDOP is a factor that expresses the expected uncertainty in determining the 3-dimensional position of the GPS based on the alignment of available satellites; a PDOP <2 is considered ideal or excellent, whereas a PDOP >10 indicates poor or bad satellite geometry.

**Figure 1:**
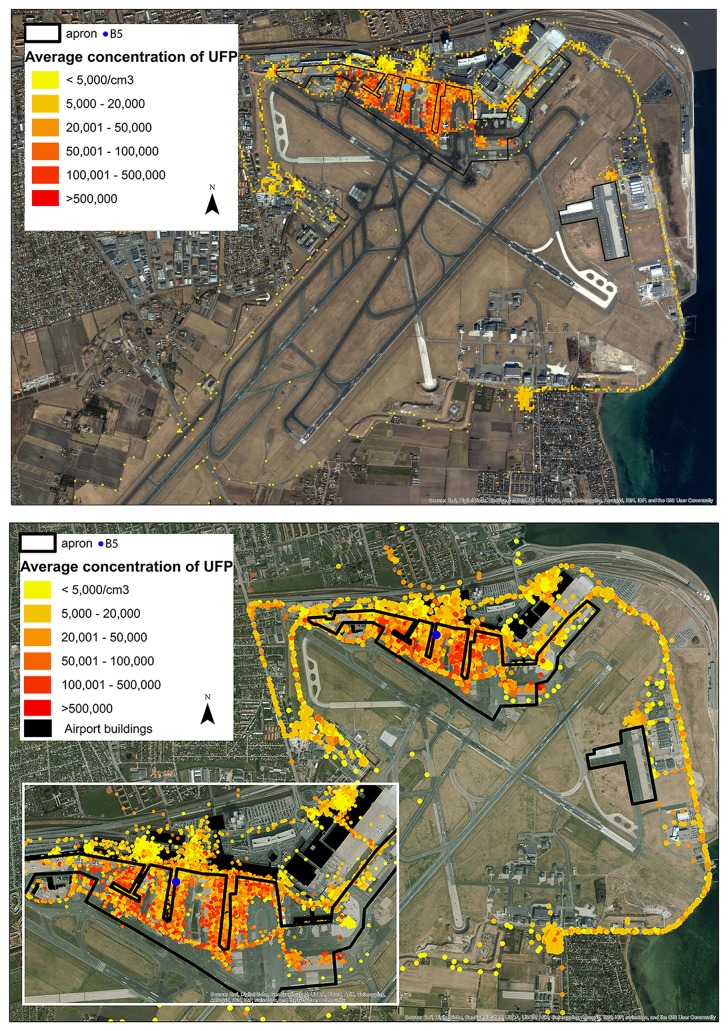
Map of all measurements of ultrafine particles per cm^3^ at Copenhagen Airport. Measurements are shown in raster cells of 25 m^2^.

The 30 employees were equipped with a NanoTracer and a GPS. The NanoTracer was fixed to a belt at the hip and was worn free of clothes. Concentrations of UFP depend on metrological conditions such as wind speed and rain. To deal with this issue and to reduce problems with peak hours at the airport, the measurements were conducted on the same day for one employee from each occupational group, in the timeframe 7.30AM-3.00PM during normal airport activities. All measurements started and ended outdoors on the apron at gate B5, see Figure 1. The measurements were carried out on 8 days distributed over 2 weeks in October 2012 (8.−11. +22.−25. October). All employees were nonsmokers and were informed to do their job as usual.

### Data processing

All GPS files were processed using the Personal Activity and Location Measurement System (PALMS), developed by the University of California, San Diego (ucsd-palms-project.wikispaces.com) [15], [22]. PALMS identified invalid data points using extreme speed or extreme changes in distance and elevation, and replaced invalid points by imputing data from the last known valid point, for up to 3 minutes. PALMS also determined if an epoch was taking place outdoors (based on the satellite to noise ratio) and if an epoch was part of a trip (defined as a continuous period of movement of at least 5 minutes, allowing for stationary periods of maximum 2 minutes). Trips were categorized into 3 modes: walking, biking and in a vehicle. Processed GPS data were then matched to the NanoTracer data based on the timestamps of each data point based on an exact time match or a match to the nearest timestamp before the NanoTracer data point in case of a timestamp miss-match. The enriched dataset, consisting of 16 sec epochs with 31,864 observations of GPS data (location, indoors/outdoors, trip mode, PDOP) and information on UFP/cm^3^ (size and number). Data were loaded into a Geographic Information System (ArcGIS 10.1) for further analysis, compilation, visualization and export into statistical analyses software (SAS 9.3). All apron areas were digitized to be able to divide data points into apron/non-apron.

### Validity of indoor-outdoor classifications in PALMS

The sensitivity and specificity of the indoor-outdoor classification in PALMS has previously been shown to be 82% and 88%, respectively [23] and another study showed sensitivity ranging from 74–100% in open air locations [22]. We are not aware of previous studies using GPS measurements inside an aircraft so the validity of how PALMS classifies GPS point inside an aircraft is unknown. To determine the validity of the PALMS classifications of being inside an aircraft a test was conducted by collecting GPS measurements for an employee from the cleaning department for 70 minutes during transportation to the aircraft, and while working inside the aircraft, in combination with completing a detailed activity log. The GPS unit was configured to log data every second. 78% of the measurements were correctly classified as inside and all measurements classified as outside the aircraft were correctly classified.

### Statistical analyses

Proportions, frequencies, medians and quartiles were calculated to describe the time each occupational group spent on the apron and outside the apron, subdivided into time spent indoors (building or aircraft), outdoors and in-vehicle.

For further analysis all observations of UFP/cm^3^ were log-transformed as data approximated a lognormal distribution. Geometric means (GM) and 95% confidence intervals (95% CI) were calculated for each occupational group. The overall differences between the five occupational groups and the pairwise comparison were assessed using a linear mixed model and 5% significance level. All analyses were adjusted for variation within individuals by including this as a random effect. Furthermore, the data were adjusted for time and day to remove potential effects of including more data from certain days or hours than from others. The time variable was based on whole hours. All analyses were performed with SAS 9.3 using proc mixed.

## Results

The overall measurement time included for each of the five occupational groups was relatively similar except for landside security, which only contributed with 6.7% of the total measurement time (Table 1). Landside security spent the smallest proportion of time on the apron (9%) while baggage handlers spent 76% of their working time on the apron. The highest concentration of UFP/cm^3^ was measured outdoors on the apron. During their stay on the apron catering drivers were exposed to the highest amount of UFP/cm^3^ with a median of 43×10^3^ UFP/cm^3^, followed by airside security (median 33×10^3^ UFP/cm^3^) and baggage handlers (median 28×10^3^ UFP/cm^3^). However, baggage handlers spent the largest amount of time on the apron and accumulated the largest total exposure during their working day. Baggage handlers and cleaning staff were exposed to the highest concentration of UFP/cm^3^ on the apron while being in-vehicle, whereas catering drivers and airside security were exposed to the highest amount of UFP/cm^3^ while working outdoors.

**Table 1:** Description of participants and measurements.

	Total N = 30	Baggage handlers (n = 6)	Catering drivers (n = 7)	Cleaning staff (n = 8)	Airside security (n = 7)	Landside security (n = 2)
**Gender**						
Male n	26	6	7	8	5	0
Female n	4	0	0	0	2	2
**Measurement in minutes mean**	293	271	301	316	279	294
**Stay on apron/non-apron in minutes (%)**						
Apron	3 849	1 231 (75.6)	738 (35.0)	1 554 (61.5)	273 (14.0)	54 (9.0)
Non-apron	4 954	396 (24.4)	1 368 (65.0)	975 (38.5)	1 680 (86.0)	535 (91.0)
**Place of stay in minutes (%)**						
**Apron**						
Indoors	1 924	669 (41.1)	137 (6.5)	955 (37.8)	110 (5.6)	54 (9.2)
Outdoors	1 643	511 (31.4)	516 (24.5)	499 (19.7)	117 (6.0)	0 (0.0)
In-vehicle	282	51(3.1)	85 (4.0)	100 (4.0)	46 (2.4)	0 (0.0)
**Non-apron**						
Indoors	2 991	351 (21.6)	351 (16.7)	727 (28.7)	1 043 (53.4)	519 (88.3)
Outdoors	1 361	35 (2.2)	792 (37.6)	134 (5.3)	384 (19.7)	16 (2.7)
In-vehicle	602	10 (0.6)	225 (10.7)	114 (4.5)	252 (12.9)	0 (0.0)
**UFP size (nm) median (min.-max.)** a	37 (17–146)	38 (18–104)	33 (19–110)	37 (17–118)	39 (18–146)	47 (27–105)
**UFP (10^3^/cm^3^) apron/non-apron median (Q1**–**Q3)**						
Apron	27 (10–67)	28 (12–71)	43 (13–110)	23 (7–51)	33 (9–99)	5 (4–6)
Non-apron	8 (3–17)	23 (9–42)	10 (6–18)	4 (1–11)	8 (4–18)	3 (2–6)
**UFP (10^3^/cm^3^) by place median (Q1**–**Q3)**						
**Apron**						
Indoors	22 (8–45)	22 (12–38)	34 (11–112)	22 (6–47)	32 (7–75)	5 (4–6)
Outdoors	37 (11–103)	45 (13–144)	50 (14–114)	23 (8–61)	48 (12–167)	N/A^b^
In-vehicle	32 (14–80)	68 (26–197)	29 (19–73)	31 (19–67)	20 (8–71)	N/A^b^
**Non-apron**						
Indoors	6 (3–14)	23 (9–40)	12 (7–17)	2 (1–6)	7 (3–17)	3 (2–6)
Outdoors	10 (5–20)	26 (12–96)	9 (6–18)	9 (3–18)	11 (6–24)	4 (4–5)
In-vehicle	11 (4–21)	N/A^b^	10 (5–20)	17 (7–26)	9 (2–17)	N/A^b^

a 1% of the measurements were >100 n**m.**

b <50 observations.

Figure 1 shows a map of all measurements of UFP/cm^3^ at Copenhagen Airport across all monitoring days and occupational groups performed in October 2012. As expected, the highest concentration of UFP/cm^3^ was measured on the apron. The concentration away from the apron hardly exceed 20×10^3^ UFP/cm^3^, thus employees working at CPH are exposed to the highest levels of UFP/cm^3^ while working on the apron.

**Figure 2:**
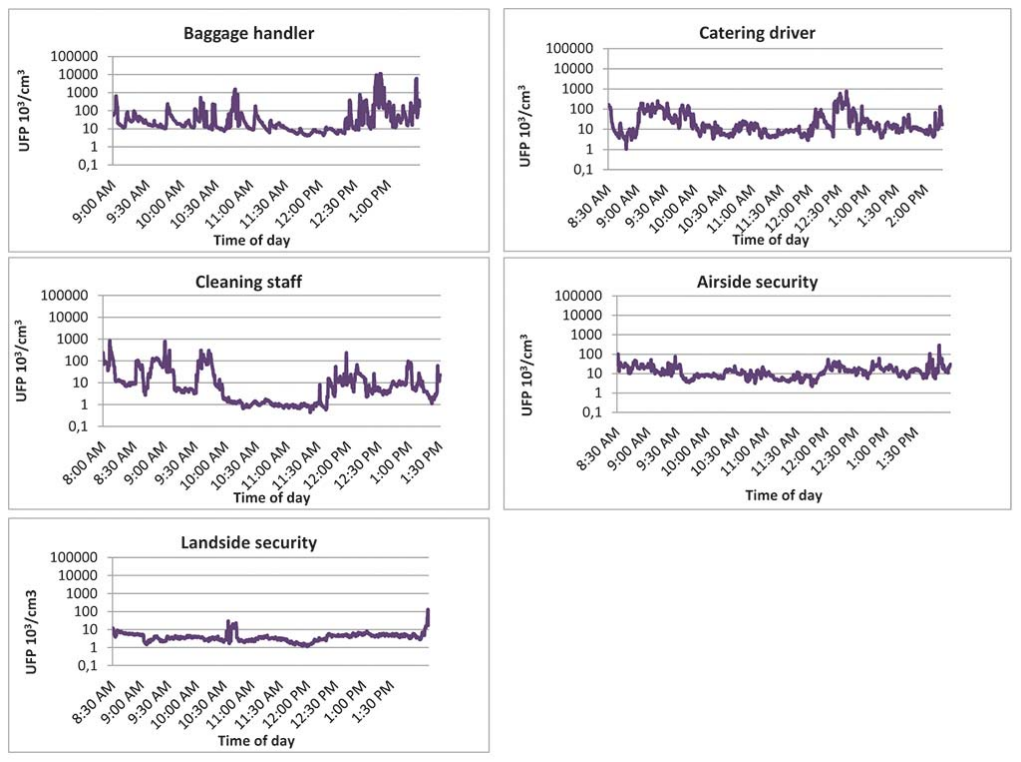
Examples of one day measurements of one employee from each occupational group.

Figure 2 shows typical examples of one day measurements of one employee from each occupational group. Figure 2 indicates that baggage handlers were exposed to the highest peak levels of UFP/cm^3^ during a typical working day, followed by catering drivers, cleaning staff, airside security and landside security.

After adjustment for variations within individuals and time and date, Table 2 shows the highest GM for baggage handlers (37×10^3^ UFP/cm^3^) and the lowest GM for landside security (5×10^3^ UFP/cm^3^). Further, catering drivers, cleaning staff and airside security had GM around the same range. In the pairwise comparisons we found that baggage handlers were significantly more exposed to UFP/cm^3^ compared to the other four groups whereas landside security were significantly less exposed compared to the other four groups. Additionally, we found no significant difference in the GM exposure levels between catering drivers, cleaning staff and airside security.

**Table 2:** Exposure to ultrafine particles among five occupational group's at Copenhagen Airport, October 2012.

Variable	Statistics	Baggage handlers	Catering drivers	Cleaning staff	Airside security	Landside security	P-value, mixed linear model
UFP 10^3^/cm^3^	GM (CI 95%) crude^a^	38 (25–56)	18 (12–25)	11 (8–15)	11 (7–15)	4 (2–8)	<0.001
UFP 10^3^/cm^3^	GM (CI 95%) adjusted^b^	37 (25–55)	20 (14–29)	12 (9–17)	12 (8–18)	5 (2–11)	<0.001

a Adjusted for variation within individuals.

b Adjusted for variation within individuals, time and date.

GM, Geometric mean.

UFP, ultrafine particles.

To test the validity of the results with respect to the precision of GPS measurements, all analysis were repeated including only those GPS points with a PDOP <2, i.e. with an ideal or excellent accuracy. The GM and p-values only changed slightly (data not shown).

## Discussion

The aim of this study was to compare the occupational exposure to UFP among five different occupational groups at CPH. We found significant higher GM in the group of baggage handlers as well as significant lower GM in the group of landside security compared to the other groups, when adjusted for variation within individuals and time and date. We found no significant difference between the three other groups (catering drivers, cleaning staff and airside security). Our results therefore indicate a division into three exposure groups with baggage handlers in a highly-exposed group, catering drivers, cleaning staff and airside security in a medium exposed group, and landside security in a low exposed group. These results are similar to exposure classifications used in two earlier studies investigating health effects at airports [11], [24]. Additionally, Yang et al. classified employees into two exposure groups with airside security in the same group as terminal workers [12]. These previous studies investigated occupational exposure at airports and the related health effects are characterized by a lack of quantitative data on occupational exposure among exposed and unexposed employees or a lack of precision in the exposure time [13]. However, the present study contributes with quantitative data from personal monitoring of employees and supports a division into three groups with high, medium and low exposure to UFP.

### Strengths and weaknesses

To our knowledge this is the first study investigating an exposure profile of UFP at an airport based on both location of the employee and personal monitoring of UFP. Data assessed from stationary measurements on the apron at CPH in October 2012 showed average concentrations at GM: 29×10^3^ UFP/cm^3^. The estimated exposure levels measured by personal exposure measurements hence showed higher levels than the background pollution on the apron at CPH, at least for baggage handlers. A further strength of this study is the design which enabled inclusion of one employee from each occupational group on the same day, which made it possibly to take variations in wind and weather into account. Because of high homogeneity in the job functions within each group the results can easily be transferred to other employees in the same occupational group.

The study also has some potential weaknesses. Firstly, the results relied on measurements in the day time and not for a whole working day, and the measurements were conducted only in autumn days. The average wind direction at CPH during October 2012 was 207° (south/south-west). At CPH most of the apron is facing south-west. The wind direction could have had an impact on the absolute levels of UFP, but it is not very likely that the relative differences between occupational groups would change by wind direction. Nice or inclement weather might influence the degree to which employees stay on the apron. However, time on the apron is mainly determined by the work tasks, which do not change by season or weather, and it is not very likely that the weather would influence staying time on the apron differently for the different occupational groups. Absolute UFP concentrations may also depend on the number and distances to passing jets and vehicles, if engines were running or not, and on the airflow in the cabins during service work. We did not have the resources to record these sources of UFP. One can only speculate about the degree to which factors may influence the relative differences between occupational groups.

Another weakness may be the precision of the NanoTracer. A previous study comparing different battery operated nanoparticle monitors found that the NanoTracer could over- and underestimate the particle number concentration with up to 30% [19]. However, this is particularly for UFP concentrations with a size above 120 nm which were in low numbers in our measurements [20]. Among all measurements only one percent of the particles were above 100 nm. Accordingly, the total number concentration is not likely to have been overestimated due to contributions from larger particle sizes.

We used GPS units to determine time and position of employees. A previous study has found a high precision of the QStarz GPS unit [15]. However, the results of time and position of employees could be a consequence of imprecise GPS data points because of buildings and pent roofs in the airport as well as working tasks inside the aircraft [15]. We found a slightly lower sensitivity than Tandon et al. and Kerr et al. found for categorizing indoors vs. outdoors in an open air environment [22], [23]. Accordingly, the classification of indoors vs. outdoors in the present study might result in misclassification in determining indoors vs. outdoors stay in aircrafts, especially among groups, which often moves from aircraft to aircraft, such as baggage handlers and cleaning staff [23]. Further, the sensitivity and specificity found in this study could be a consequence of few measurements included in the validity test. Consequently, the validity of the GPS/PALMS algorithms should be further tested in more areas of the airport and for a longer time period in future studies.

In conclusion this study demonstrates a strong gradient of exposure to UFP in ambient air across occupational groups of airport employees. This information may be utilized in a job exposure matrix to study adverse health effects from exposure to UFP and will be used accordingly in an ongoing epidemiological study of the risk of the UFP-related cardiovascular disease among employees at the airport.
